# Evaluating antimicrobial effectiveness in acute uncomplicated cystitis: A retrospective single‐center study

**DOI:** 10.1002/jgf2.70034

**Published:** 2025-05-22

**Authors:** Takuhisa Nukaya, Kiyohito Ishikawa, Ryoichi Shiroki

**Affiliations:** ^1^ Department of Urology Fujita Health University School of Medicine Nagoya Aichi Japan

**Keywords:** cephalosporins, cystitis, *E. coli*, levofloxacin, β‐lactam/β‐lactamase inhibitor

## Abstract

**Background:**

Acute uncomplicated cystitis (AUC) is a urinary tract infection and is generally treated using antimicrobial therapy. *Escherichia coli* is the main causative agent of AUC. Recently, the prevalence of fluoroquinolone (FQ)‐resistant‐*E. coli* has demonstrated a noticeable increase. In this study, we aimed to investigate the effectiveness of appropriate antimicrobial treatment in AUC caused by *E. coli* in real‐world clinical settings.

**Methods:**

This retrospective cohort study reviewed the records of patients with AUC treated at the urology department of Minami Cooperative Hospital between April 2016 and December 2020. Effectiveness was defined as clinical improvement.

**Results:**

The study cohort of 730 patients had a median age of 65.5 years (interquartile range, 57–78 years) and 23.2% were aged <55 years. *E. coli* was detected in 73.4% of patients, of whom 26.7% had levofloxacin (LVFX)‐resistant strains. LVFX‐resistant *E. coli* was associated with age ≥55 years and recurrent cases. Effectiveness was determined in 75.1% of cases, of which 75% complied with the Japanese or other international guidelines. The overall treatment effectiveness was highest with β‐lactam (BL)/β‐lactamase inhibitor (BLI) combinations (94.7%). The effectiveness of first‐ and third‐generation cephalosporins (CPs) was 81.1–83.3%, and that of FQs and sulfamethoxazole–trimethoprim (ST) was 82.6–83.8%. For LVFX‐resistant *E. coli*, the treatment effectiveness was highest (100%) with BL/BLI combinations, intermediate (75–81%) with first‐ and third‐generation CPs and ST, and lowest (50%) with FQs.

**Conclusions:**

BL/BLI combinations had the highest effectiveness for the treatment of AUC.

## 1 INTRODUCTION

Acute uncomplicated cystitis (AUC) is a urinary tract infection that commonly affects female patients and is generally responsive to antimicrobial therapy. *Escherichia coli* is the major causative agent of AUC, and in recent years an increase in the prevalence of fluoroquinolone (FQ)‐resistant‐*E. coli* has been reported.^1^, ^2^, ^3^ The Japanese Association for Infectious Diseases and Japanese Society of Chemotherapy (JAID/JSC) Guide to the treatment of infectious diseases (revised in 2023)^4^ recommends β‐lactam (BL)/β‐lactamase inhibitor (BLI) combinations (BL/BLI combinations) such as clavulanic acid/amoxicillin (CVA/AMPC) and sulbactam/ampicillin (SBT/ABPC) as first‐line treatment of AUC, irrespective of whether the patients are pre‐ or postmenopausal, especially when the causative organism is unknown. This study aimed to evaluate the effectiveness of antimicrobial treatment of AUC using real‐world clinical data.

## 2 METHODS

### 2.1 Study design and participants

This retrospective study analyzed the data of 730 patients who were treated at the urology department of Minami cooperative Hospital in Nagoya City between April 2016 and December 2020 and presented with any of the following: frequent urination, pain during urination, urinary urgency, or lower abdominal pain, with ≥5 white blood cells/high power field in centrifuged urine sediment and a clinical diagnosis of AUC with a single detectable organism on culture. Among 730 patients with positive urine culture, the effectiveness of treatment was determined in 548 who re‐visited within 5–14 days of antimicrobial prescription. Male patients, those with underlying diseases such as stones or malignancies in the urinary tract, and those undergoing treatment with anti‐cancer drugs were excluded from the analysis. The choice of antimicrobial therapy and the duration of antimicrobial administration were made at the discretion of the treating physician. The clinical response to treatment was judged to be effective if all symptoms disappeared or improved to levels equal to those preceding the onset of infection based on the guidelines for conducting clinical trials on urogenital infections.^5^

### 2.2 Microbiological investigations

Midstream clean‐catch urine samples were sent to the laboratory for standard urinalysis and culture. Bacterial growth of ≥10^4^ colony‐forming units (CFUs)/mL urine was considered positive. Bacterial identification and drug susceptibility tests were performed using the matrix‐assisted laser desorption Bio‐Typer (Bruker Daltonics GmbH & Co. KG, Bremen, Germany), DPS MIC 192/ID microbial susceptibility analyzer (Eiken Chemical, Ishikawa, Japan), and Dry Plate Eiken (Eiken Chemical, Tochigi, Japan). Drug susceptibility testing against *E. coli* was performed using ampicillin (ABPC), piperacillin (PIPC), cefaclor (CCL), cefazolin (CEZ), cefotiam (CTM), cefotaxime (CTX), ceftazidime (CAZ), cefpirome (CPR), cefpodoxime proxetil (CPDX‐PR), cefmetazole (CMZ), flomoxef (FOMX), imipenem/cilastatin (IPM/CS), meropenem (MEPM), aztreonam (AZT), clavulanic acid/amoxicillin (CVA/AMPC), sulbactam/cefoperazone (SBT/CPZ), gentamicin (GM), amikacin (AMK), minocycline (MINO), fosfomycin (FOM), levofloxacin (LVFX), and sulfamethoxazole‐trimethoprim (ST). The BL/BLI combinations used were CVA/AMPC and SBT/ABPC.

The appropriate antimicrobial agents and durations of administration according to the 2023 JAID/JSC Infectious Diseases Guide^4^ and the 2019 American Urological Association Guideline 2019^6^ were 7 days for BL/BLI and first‐generation CPs, 3–7 days for third‐generation CPs, 3 days for FQs, and 3 days for ST. The treatment regimens described above were defined as the appropriate use of antimicrobial agents.

### 2.3 Statistical analyses

Logistic regression was used for univariate and multivariable analyses to identify risk factors for the development of FQ‐resistant *E. coli*. Two‐sided *p‐*values <0.05 were considered statistically significant. All statistical analyses were performed using EZR, a graphical user interface for R (The R Foundation for Statistical Computing, Vienna, Austria).^7^

## 3 RESULTS

### 3.1 Patient background, causative organisms, and drug susceptibility results for *E. coli*

The mean age of 730 patients with clinically diagnosed cystitis and identified causative organisms was 65.5 years; 169 (23.2%) were aged <55 years and 561 (76.8%) were aged ≥55 years. The causative bacteria were *E. coli* in 536 cases (73.4%), *Klebsiella* spp. in 63 cases (8.6%), and *Enterococci* in 22 cases (3.0%).

**FIGURE 1:**
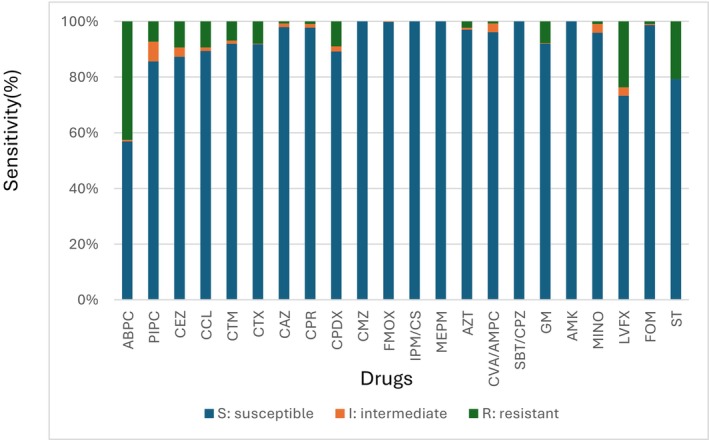
Drug susceptibility results for *Escherichia coli*. Sensitivity of *E. coli* to ABPC was 56.9%, PIPC 85.7%, CEZ 87.4%, CCL 89.4%, CTM 91.9%, CTX 91.8%, CAZ 97.9%, CPR 97.8%, CPDX 89.2%, CMZ 100%, FMOX 99.8%, IPM/CS 100%, MEPM 100%, AZT 97.0%, CVA/AMPC 96.1%, SBT/CPZ 100%, GM 92.0%, AMK 100%, MINO 95.9%, LVFX 73.7%, FOM 98.7%, and ST 79.3%. ABPC, ampicillin; AMK, amikacin; AZT, aztreonam; CAZ, ceftazidime; CCL, cefaclor; CEZ, cefazolin; CMZ, cefmetazole; CPDX‐PR, cefpodoxime proxetil; CPR, cefpirome; CTM, cefotiam; CTX, cefotaxime; CVA/AMPC, clavulanic acid/amoxicillin; FOM, fosfomycin; FOMX, flomoxef; GM, gentamicin; I, intermediate; IPM/CS, imipenem/cilastatin; LVFX, levofloxacin; MEPM, meropenem; MINO, minocycline; PIPC, piperacillin; R, resistant; S, susceptible; SBT/CPZ, sulbactam/cefoperazone; ST, sulfamethoxazole‐trimethoprim.

The sensitivity of *E. coli* to antimicrobial agents was as follows: ABPC 56.9%, PIPC 85.7%, CCL 89.4%, CEZ 87.4%, CTM 91.9%, CTX 91.8%, CAZ 97.9%, CPR 97.8%, CPDX 89.2%, CMZ 100%, FOMX 99.8%, IPM/CS 100%, MEPM 100%, AZT 97.0%, CVA/AMPC 96.1%, SBT/CPZ 100%, MINO 95.9%, GM 92.0%, AMK 100%, FOM 98.7%, LVFX 73.3%, and ST 79.3%. The incidence of LVFX‐resistant *E. coli* was 26.7% (Figure 1).

### 3.2 Selection of antimicrobial agents

Of the 730 patients diagnosed with AUC, 151 (20.7%) were treated with BL/BLI combinations. Of these, 264 (36.1%) were treated with CPs: 69 (9.5%) with first‐generation CPs and 195 (26.7%) with third‐generation CPs. Moreover, 182 (24.9%) were treated with FQs, 115 (15.8%) were treated with ST, and 12 (1.64%) were treated with FOM.

The antimicrobial treatment was appropriate in 94.7% of patients (143/151) treated with BL/BLI combinations, 100% of patients (69/69) treated with first‐generation CPs, 99.5% of patients (194/195) treated with third‐generation CPs, 26.1% of patients (48/182) treated with FQs, and 86.1% of patients (99/115) treated with ST.

### 3.3 Effectiveness of antimicrobial agents in patients with appropriate use

Of the 730 patients with clinically diagnosed AUC, the clinical effectiveness of antimicrobial therapy was determined by re‐examination in 548 patients (75.1%). The mean time to re‐examination was 9.1 days. Antimicrobials were used appropriately in 411/548 patients (75.0%) and the treatment was clinically effective in 95.4% of patients (104/109) treated with BL/BLI combinations: 83.3% of patients (45/54) treated with first‐generation CPs, 82.1% of patients (106/129) treated with third‐generation CPs, 84.8% of patients (28/33) treated with FQs, and 83.7% of patients (72/86) treated with ST.

**FIGURE 2:**
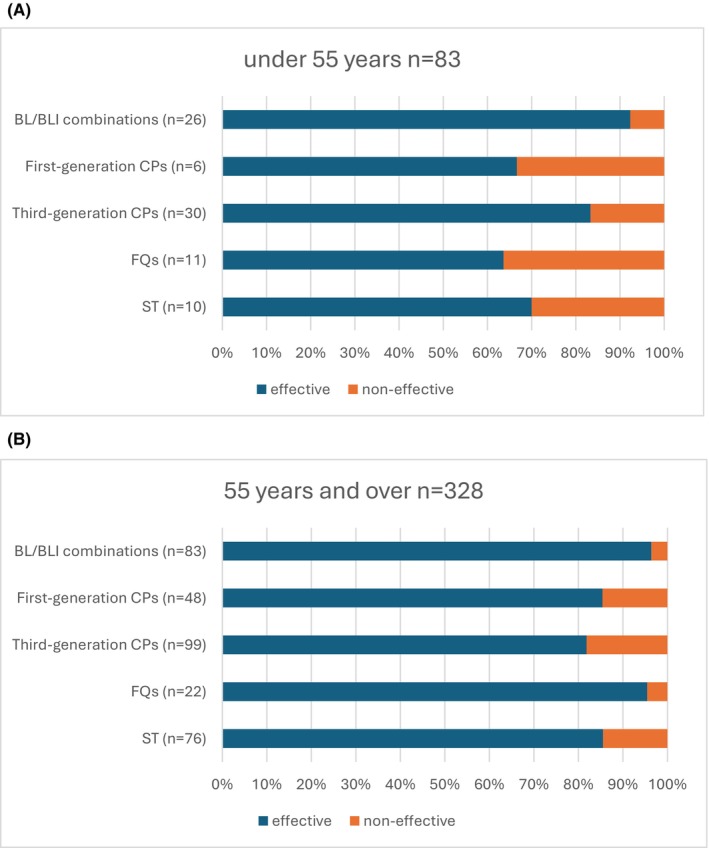
Effectiveness of the appropriate use of antimicrobial agents in AUC. (A) For patients <55 years, the effectiveness was 92.3% for BL/BLI combinations, 66.7% for first‐generation CPs, 83.3% for third‐generation CPs, 63.6% for FQs, and 70% for ST. (B) For patients ≥55 years, the effectiveness was 96.3% for BL/BLI combinations, 85.4% for first‐generation CPs, 81.8% for third‐generation CPs, 95.5% for FQs, and 85.5% for ST. AUC, acute uncomplicated cystitis; BL/BLI, β‐lactam/β‐lactamase inhibitor; CPs, cephalosporins; FQs, fluoroquinolones; ST, sulfamethoxazole–trimethoprim.

Of the 411 patients with appropriate antimicrobial use, 83 (20.2%) patients were aged <55 years, and 328 (79.8%) were aged ≥55 years, using age as a proxy for menopausal status. In those aged <55 years, the effectiveness was 92.3% (24/26) for BL/BLI combinations, 66.7% (4/6) for first‐generation CPs, 83.3% (25/30) for third‐generation CPs, 63.6% (7/11) for FQs, and 70% (7/10) for ST. Among those aged ≥55 years, the effectiveness was 96.3% (80/83) for BL/BLI combinations, 85.4% (41/48) for first‐generation CPs, 81.8% (81/99) for third‐generation CPs, 95.5% (21/22) for FQs, and 85.5% (65/76) for ST (Figure 2).

### 3.4 Efficacy of antimicrobial agents against LVFX‐resistant *E. coli*

**FIGURE 3:**
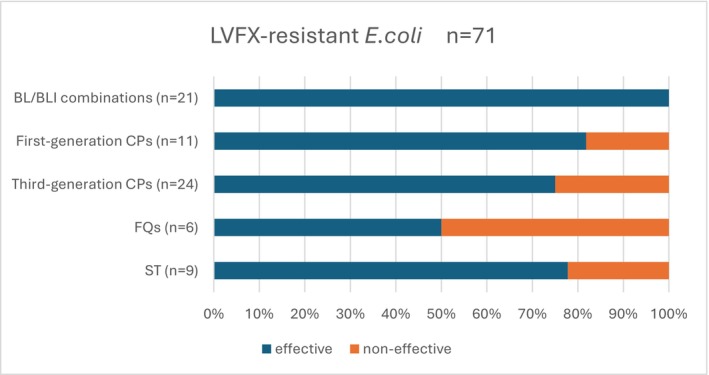
Effectiveness of antimicrobials against LVFX‐resistant cephalosporins. The effectiveness was 100% for BL/BLI combinations, 81.8% for first‐generation CPs, 75% for third‐generation CPs, 50.0% for FQs, and 77.8% for ST. AUC, acute uncomplicated cystitis; BL/BLI, β‐lactam/β‐lactamase inhibitor; CPs, cephalosporins; FQs, fluoroquinolones; LVFX, levofloxacin; ST, sulfamethoxazole–trimethoprim.

Of the 143 patients in whom LVFX‐resistant *E. coli* was detected, antimicrobials were used appropriately, and the clinical effectiveness could be determined in 71 cases (49.7%): Treatment was effective in 100% of patients (21/21) treated with BL/BLI combinations, 81.8% of patients (9/11) treated with first‐generation CPs, 75% of patients (18/24) treated with third‐generation CPs, 50.0% of patients (3/6) treated with FQs, and 77.8% of patients (7/9) treated with ST (Figure 3).

### 3.5 Risk factors for LVFX‐resistant *E. coli*

The number of cases with LVFX‐resistant *E. coli* was 24/126 (19.1%) in 2016, 33/153 (21.5%) in 2017, 30/174 (17.2%) in 2018, 36/197 (18.2%) in 2019, and 20/80 (25.0%) in 2020. The frequency by age was: 20–29 years (2/33; 6.1%), 30–39 years (9/46; 19.5%), 40–49 years (6/65; 9.2%), 50–59 years (5/64; 7.8%), 60–69 years (31/131; 23.6%), 70–79 years (56/239; 23.4%), 80–89 years (29/138; 21.1%), and ≥90 years (5/14; 35.7%). The incidence of LVFX‐resistant *E. coli* infection was markedly higher in the 30–39 years age group and among those aged ≥60 years. In addition, single variable analysis of risk factors for LVFX‐resistant *E. coli* infection showed that patients aged ≥55 years and patients with recurrent AUC were more likely to have LVFX‐resistant infection. In the multivariable analysis, age ≥ 55 years was an independent risk factor for LVFX‐resistant infection (Table 1).

**TABLE 1:** Risk factors for development of levofloxacin‐resistant *Escherichia coli* infection.

	Unadjusted analysis			Multivariable analysis		
	Odds ratio	95% CI	*p*‐value	Odds ratio	95% CI	*p*‐value
Age ≥ 55 years	2.47	1.43–4.24	0.001	2.34	1.36–4.05	0.002
Recurrent	1.58	1.02–2.44	0.039	1.4	0.9–2.17	0.13
Urinary sugar	1.69	0.81–3.54	0.168			
Urinary occult blood	0.74	0.46–1.18	0.21			
Nitrite	1.24	0.74–2.06	0.41			
History of LVFX use	1.80	0.85–3.82	0.12			

*Note*: Univariate analysis showed significant differences among patients aged ≥55 years and recurrent cases; age ≥55 years was an independent factor in the multivariate analysis. Results of qualitative and semi‐quantitative urinalysis: urinary sugar (+ to +++); urinary occult blood (+ to +++); nitrite (+ to +++).

Abbreviations: CI, confidence interval; LVFX, levofloxacin.

The annual proportion of ESBL‐producing *E. coli* showed no significant variation by year, with rates of 3.2% (4/126 cases) in 2016, 8.5% (13/153 cases) in 2017, 4.0% (7/174 cases) in 2018, 7.6% (15/197 cases) in 2019, and 7.5% (6/80 cases) in 2020. The incidence of ESBL‐producing *E. coli* did not show any substantial variation by age. It was 9.1% (3/33 cases) in patients aged 20–29 years, 2.2% (1/46 cases) in those aged 30–39 years, 4.6% (3/65 cases) in those aged 40–49 years, 4.6% (3/64 cases) in those aged 50–59 years, 10.7% (14/131 cases) in those aged 60–69 years, 5.9% (14/239 cases) in those aged 70–79 years, 4.3% (6/138 cases) in those aged 80–89 years, and 7.1% (1/14 cases) in individuals aged 90 years and older.

The LVFX resistance rate among ESBL‐producing *E. coli* was 68.8% (31/45); however, high susceptibility rates were observed for ABPC/SBT (100%, 45/45), MINO (93.3%, 42/45), and FOM (100%, 45/45).

## 4 DISCUSSION

AUC is more common in sexually active women and middle to older adulthood, particularly around the time of menopause. The mean age in this study was 65.8 years, making it a condition observed predominantly in older women. Gram‐positive bacteria accounted for 8.6% and gram‐negative bacteria accounted for 91.4% of isolates (*E. coli* in 73.4%; *Klebsiella* spp. in 8.6%). Surveillance conducted in Japan in 2010, 2015, and 2018 reported a detection rate for *E. coli* of ~70% in postmenopausal women,^8^, ^9^, ^10^ which is consistent with the results of this study. In premenopausal women, the detection rates of gram‐positive cocci and *Staphylococcus saprophyticus* were 13.4 and 5%, respectively, in 2010; 27.2 and 11.1%, respectively, in 2015; and 22.1 and 7.5%, respectively, in 2018.^8^, ^9^, ^10^ No cases of *S. saprophyticus* infection were detected in this study. This may be because some cases of *S. saprophyticus* infection being classified as coagulase‐negative staphylococci by the laboratory analyzer. Among the participants in this study, gram‐positive cocci were detected in only a small percentage (15.0%) of those aged <55 years, which is lower than the incidence reported in previous studies; however, the results in patients aged ≥55 years were similar to those reported in previous studies.^8^, ^9^, ^10^ In this study, the susceptibility results for *E. coli* were 96.1% for BL/BLI, 73.3% for LVFX, and 79.3% for ST. In another similar study,^10^ the susceptibility results were 78.6–81.0% for BL/BLI, 84.4% for LVFX, and 85.7% for ST. There were no differences in the sensitivity results for other drugs between the two studies; thus, the participants in our study had high susceptibility to BL/BLI combinations and only moderate susceptibility to LVFX and ST.

In this study, the antimicrobials were selected at the discretion of the attending physician. CPs were the most frequently selected antimicrobial agents (36.1%), with BL/BLI combinations being used in 20.7%, FQs being used in 24.9%, and ST being used in 15.8% of patients. The relatively low use of FQs is likely owing to physicians being aware of the rise in the incidence of FQ‐resistant *E. coli* infections and the antimicrobial resistance (AMR) action plan. Of the patients prescribed FQs, it was prescribed for more than 3 days for the majority (73.9%), contrary to the guidelines, and long‐term administration was common, whereas the other antimicrobials were generally used appropriately. In Japan, where access to outpatient clinics is easy, a high proportion of patients with AUC return to the clinic after 7 days to determine the effectiveness of treatment. This may have resulted in the administration of FQs for longer than the recommended 3 days. According to a prescription‐based study conducted in Japan,^11^ antimicrobial agents were prescribed to 98% of patients with AUC; penicillin or BL/BLI combinations were prescribed to 23.7%, first and second‐generation CPs to 3.5%, third‐generation CPs to 31%, FQs to 30%, and ST to 2.2%. The proportion of patients prescribed ST was significantly lower than that in this study. In other countries, ST has become the first‐line treatment for AUC; however, its long‐term use has led to increased resistance rates of 14.6–60% in Europe, 18% in the USA, and 3.9–15% in Japan.^3^ STs are not included in the Japanese guidelines because they cause skin and gastrointestinal symptoms in <0.1–5% of patients and have complicated drug interactions; however, they are recommended in international guidelines, which is why the prescription rate was high in this study.^12^, ^13^, ^14^ The effectiveness in this study was 85.5% for patients aged ≥55 years, making it suitable for use in Japan in patients aged ≥55 years.

Nitrofurantoin, which is recommended as a first‐line drug in the international guidelines, is reported to be less likely to induce bacterial resistance than treatment with FQs,^3^, ^15^ and is expected to be approved in Japan. The 2023 European Association of Urology guidelines^16^ recommend oral CPs for 3–7 days as second‐line therapy; although some meta‐analyses have reported that single dosing is possible.^13^, ^17^ In this study, the effectiveness of third‐generation CPs was 83.3% in patients aged <55 years and 81.8% in patients aged ≥55 years, suggesting that they can be used regardless of age. Use of narrow‐range first‐generation CPs is also recommended to prevent the emergence of drug resistance, and their use is likely to increase in the future because of reports of their effectiveness.^14^, ^18^ The results of this study suggest that first‐generation CPs are 85.4% effective in patients aged ≥55 years and can be used in patients with AUC in this age group. Despite the low prevalence of gram‐positive cocci in this study and the limited number of cases in patients aged <55 years, BL/BLI combinations, and third‐generation CPs are recommended for AUC in patients aged <55 years because their effectiveness is more than 80%. In patients aged ≥55 years, LVFX should be administered with caution as a result of the increased risk of LVFX resistance; however, in this study, all recommended drugs were found to have an effectiveness of ≥80% and may be considered acceptable for use.

Of 536 *E. coli* cases detected, LVFX‐resistant *E. coli* was diagnosed in 26.7% and the isolation frequency of FQ‐resistant *E. coli* derived from the urinary tract has increased over time, ranging from 6.4 to 15.6% in AUC.^8^, ^9^, ^10^ In contrast to the findings of this study, previous studies have found a high prevalence of fluoroquinolone‐resistant *E. coli* in postmenopausal women,^2^ and the increasing incidence of antimicrobial‐resistant bacterial infection is a worldwide problem, not restricted to Japan.

Although the effectiveness of first‐generation CPs against LVFX‐resistant *E. coli* has been reported to be 100%,^18^ it was 81.8% in this study. Risk factors for the detection of FQ‐resistant *E. coli* include two or more episodes of cystitis within 1 year, complicated cystitis, prior FQs therapy within 3 months, and age ≥75 years.^1^, ^19^ In this study, age ≥ 55 years and recurrent cases were risk factors for the development of LVFX‐resistant *E. coli* AUC, with age ≥ 55 years being the strongest independent risk factor. Considering that the probability of LVFX‐resistant *E. coli* infection increases with age, the use of FQs should be avoided, especially in patients aged ≥ 55 years and in recurrent cases, and BL/BLI combinations or first‐generation CPs should be used instead. However, the number of cases investigated in this study was small; therefore, further research is needed.

The effectiveness of LVFX against LVFX‐resistant bacteria can be attributed to the following reasons: The maximum blood concentration (*C*_max_) and the maximum urinary concentration (*U*_max_) following a single 500 mg dose of LVFX have been reported as 6.1 and 406 μg/mL, respectively, and the urinary concentration of the antibacterial agent is reported to be approximately 100 times higher than the blood concentration.^20^ The Clinical and Laboratory Standards Institute breakpoints widely used internationally are based on data from bloodstream infections. Based on this, the bacteria are determined to be resistant; however, as mentioned above, *U*_max_ is approximately 100 times that of *C*_max_, so even drug‐resistant bacteria are likely to be susceptible to the antibiotic to a certain extent owing to the high *U*_max_.

This study has several limitations. It was a single‐center, short‐term study; the number of cases was small; 25% of cases had no data about clinical effectiveness determination; the number of patients aged <55 years was small; the doses of antimicrobial agents were not standardized; and there were few cases of appropriate LVFX use.

Constant use of the same antimicrobial agents for the treatment of AUC may lead to increased bacterial resistance and treatment failure.^21^ The 2023 JAID/JSC Guide to Infectious Diseases recommends that therapeutic agents should be selected by estimating the causative organisms based on patient background, medical history, and Gram stain results. The measures recommended in the guidelines, including AMR countermeasures, are very useful. The BL/BLI combinations recommended in the JAID/JSC Guide to Infectious Diseases 2023 showed the highest effectiveness in this study. Additionally, in patients with AUC aged <55 years, BL/BLI combinations and third‐generation CPs showed the highest effectiveness, and in patients aged ≥55 years, all recommended drugs were effective. However, FQs should be administered with caution because of the high risk of LVFX resistance. The incidence of LVFX‐resistant *E. coli* AUC increases with age; therefore, BL/BLI combinations or first‐generation CPs should be selected, taking risk factors for resistance into consideration.

## AUTHOR CONTRIBUTIONS

**Takuhisa Nukaya:** Conceptualization; writing – original draft; investigation; methodology; software; data curation; formal analysis; validation; funding acquisition; project administration. **Kiyohito Ishikawa:** Writing – review and editing; supervision. **Ryoichi Shiroki:** Supervision; writing – review and editing.

## FUNDING INFORMATION

This study did not receive any specific grants from public, commercial, or nonprofit funding agencies.

## CONFLICT OF INTEREST STATEMENT

The authors have stated explicitly that there are no conflicts of interest in connection with this article.

## ETHICS STATEMENT

Ethics approval statement: The protocols used for this research have been approved by the Institutional Review Board of Fujita Health University School of Medicine (admission number: HM24‐162). All procedures followed the ethical standards set by the responsible committee for human experimentation at both the institutional and national levels, and it conforms to the provisions of the Declaration of Helsinki and its subsequent revisions. The requirement for informed consent was waived because this was a retrospective study.

Patient consent statement: The requirement for informed consent was waived because this was a retrospective study.

Clinical trial registration: None.

## Data Availability

The datasets generated and analyzed during the current study are not publicly available owing to our hospital policy but are available from the corresponding author on reasonable request.
